# Molecular Dynamics Study on the Interfacial Properties of Short Kevlar Fiber Reinforced Polyphenylene Sulfide Composites

**DOI:** 10.3390/polym18161965

**Published:** 2026-08-12

**Authors:** Zebei Mao, Ziping Li, Danyang Liu, Jiqiang Wang, Xingkeng Shen

**Affiliations:** 1 Qianwan Institute of CNITECH, Ningbo 315336, China; maozebei@nimte.ac.cn (Z.M.);; 2 Ningbo Institute of Materials Technology and Engineering, Chinese Academy of Sciences, Ningbo 315201, China

**Keywords:** Kevlar, polyphenylene sulfide (PPS), molecular dynamics (MD), interface simulation, composite, mechanical properties

## Abstract

Polyphenylene sulfide (PPS) is a high-performance thermoplastic engineering material known for its excellent chemical resistance, thermal stability, and flame retardancy. In this work, the interfacial mechanical behavior of short-cut Kevlar fiber-reinforced PPS composites was systematically investigated by all-atom molecular dynamics (MD) simulations. By constructing a full-atom interface model between an amorphous PPS matrix and a Kevlar crystal, interfacial normal tension and tangential shear simulations were performed to reveal the mechanisms of load transfer, damage initiation, and damage evolution at the molecular scale. The results show that the interfacial normal tensile strength (approximately 245 MPa) is lower than the bulk tensile strength of pure PPS (approximately 270 MPa), which is attributed to the stiffness mismatch at the interface induced by the high modulus of Kevlar fibers, promoting the preferential initiation and propagation of voids near the geometrical interface. The tangential shear process exhibits pronounced stick-slip characteristics, with the interfacial binding energy fluctuating periodically with shear displacement, corresponding to the alternating establishment and rupture of non-bonded interactions between molecular chains. This study provides a theoretical basis for the micromechanical design of high-performance thermoplastic composite interfaces and identifies molecular-level optimization directions for future interfacial modification strategies of Kevlar/PPS systems.

## 1. Introduction

Polyphenylene sulfide (PPS) is a semi-crystalline high-performance thermoplastic polymer that has garnered significant attention due to its outstanding comprehensive properties. PPS exhibits a long-term service temperature of up to 200–240 °C, a glass transition temperature (Tg) of approximately 85–90 °C, and a melting point (Tm) of about 280–285 °C [1,2,3]. Additionally, PPS possesses excellent chemical resistance, inherent flame retardancy, good dimensional stability, and superior electrical insulation properties. These characteristics make PPS widely applicable in automotive engine compartment components, chemical filtration fabrics, electronic packaging materials, aerospace structural parts, and precision mechanical components [4,5].

However, the elastic modulus and strength of pure PPS remain insufficient for more demanding structural applications. Common modification strategies include compounding with inorganic fillers [6,7], blending with other polymers [8], and fiber reinforcement [9,10]. Among these, fiber reinforcement is widely adopted due to its high efficiency and ease of operation.

Kevlar fiber, developed by DuPont in 1965, is a high-performance organic fiber chemically known as poly(p-phenylene terephthalamide) (PPTA) [11]. Kevlar fibers are renowned for their extremely high specific strength, high specific modulus, excellent impact resistance, and good thermal stability [12]. Research on the application of Kevlar fibers in composites dates back to the 1970s, and they are now widely used in bulletproof vests, aerospace structures, sporting goods, tire cords, and optical fiber cable reinforcements [13].

Systematic investigations have been conducted on Kevlar fiber-reinforced thermoplastic polymers [14,15]. In recent years, numerous studies have shown that short Kevlar fibers can significantly improve the elastic modulus and dimensional stability of thermoplastic polymer composites. However, the reinforcement effect is highly dependent on the load transfer efficiency at the fiber/matrix interface; insufficient interfacial bonding typically leads to fiber pull-out rather than fiber fracture. Jiang et al. reviewed the reinforcement mechanisms of short fiber-reinforced thermoplastic composites, pointing out that the quality of the fiber/matrix interface is a critical factor determining the mechanical properties of the composite [16]. Jin et al. investigated interfacial strengthening techniques for fiber-reinforced thermoplastic composites and found that enhancing the interfacial shear strength can effectively improve load transfer and suppress fiber pull-out [17]. Periasamy et al. summarized interfacial engineering methods for thermoplastic composites, suggesting that interfacial modification can significantly enhance the bonding between fibers and matrix [18]. Xu et al. reviewed the research progress on Kevlar fiber-reinforced polymer composites, highlighting that interface optimization is key to improving the reinforcement effect of Kevlar [19,20].

The interface between the fiber and matrix in composites is the critical region for load transfer, directly determining the macroscopic mechanical properties of the composite. Interface theories mainly include mechanical interlocking theory, adsorption/wetting theory, chemical bonding theory, and diffusion theory [18]. For the Kevlar/PPS system, since both are aromatic polymers (PPS backbone contains benzene rings and sulfur bonds, Kevlar backbone contains benzene rings and amide bonds), a certain degree of interfacial interaction can theoretically be formed through π–π stacking and van der Waals forces. However, the smooth and chemically inert surface of Kevlar fibers limits mechanical interlocking and chemical bonding at the interface [21].

In experimental studies, Alsaadi et al. compared the interlaminar shear properties of carbon, glass, and Kevlar fabric/epoxy composites, finding that the interlaminar shear strength of Kevlar composites was lower than that of carbon fiber composites, with fracture characterized by fiber pull-out and interface debonding, indicating weak Kevlar/resin interfacial bonding [22]. McCarthy et al. reviewed test methods for interfacial shear strength in continuous fiber composites, pointing out that interfacial shear strength determines load transfer capability; when interfacial bonding is insufficient, composites are more prone to fiber pull-out rather than fiber fracture [23]. Teklal et al. investigated fiber pull-out models and fiber/matrix interfacial adhesion mechanisms, finding that fiber pull-out length, interfacial shear strength, and interfacial adhesion properties jointly determine the failure mode of composites, with weak interfaces typically characterized by smooth fiber surfaces and minimal resin residue [24].

Molecular dynamics (MD) simulation is a powerful tool for studying the microscopic mechanical behavior of composite interfaces, capable of revealing structural evolution, interaction energy changes, and mechanical response mechanisms at the atomic/molecular scale. In recent years, MD simulations have been successfully applied to the study of various composite interfaces. Yan et al. studied the interfacial microstructure and interfacial shear strength of continuous carbon fiber/polyimide composites using MD simulations, revealing the effects of different surface functional groups on interfacial interaction energy, interfacial bonding strength, and load transfer capability, achieving atomic-scale interpretation of interfacial reinforcement mechanisms [25]. Huang et al. systematically reviewed characterization methods for interfacial properties of fiber-reinforced polymer composites, pointing out that molecular dynamics simulations can reveal structural evolution, interfacial interactions, and stress transfer mechanisms at the fiber/matrix interface, and can be combined with finite element methods for multiscale interface analysis [26]. Yuan et al. reviewed the latest characterization techniques for interfaces in carbon fiber-reinforced polymer composites, pointing out that molecular dynamics simulations can be used to calculate interfacial binding energy, interfacial shear strength, and interfacial failure processes, providing a theoretical basis for interface design and performance optimization [27]. Sattar et al. combined molecular dynamics simulations with experimental methods to study the molecular structure and dynamic behavior of polymer interfaces, elucidating the mechanisms by which molecular chain conformation, interfacial interactions, and segmental motion contribute to improved interfacial performance [28]. Lubineau et al. reviewed interface toughening mechanisms in composites, pointing out the importance of understanding energy dissipation processes at interfaces, with MD simulations providing fundamental insight at the molecular level [29].

However, there is currently a lack of work that directly couples high-precision molecular models of Kevlar fibers with polymer matrices to study the interfacial mechanical behavior of Kevlar/PPS at the full-atom scale, particularly with regard to the microscopic failure mechanisms under interfacial normal and shear loading. To address this gap, this study constructs a full-atom MD model of the PPS/Kevlar interface to simulate the interfacial microstructure. Through interfacial normal tension and shear simulations, the mechanical response and damage evolution mechanisms of the interface under different loading modes are revealed.

## 2. Simulation Methods and Model Construction

### 2.1. Force Field Selection and Simulation Parameters

This study employs the GROMOS 54A7 all-atom force field as the simulation framework [30,31]. GROMOS belongs to a mature family of fixed-charge condensed-phase force fields and has demonstrated moderate yet adequate capability in describing condensed phases in benchmark comparisons with other mainstream force fields, making it a suitable general-purpose model for analyzing interfacial interactions and trends. In the GROMOS 54A7 force field, aromatic hydrogen atoms are explicitly represented, a treatment that is crucial for simulating aromatic polymers such as Kevlar and PPS, as it enables a more accurate description of the contribution of aromatic ring hydrogen atoms to non-bonded interactions [32].

Within the GROMOS force field framework, van der Waals interactions between molecules are described using the 12-6 Lennard-Jones (LJ) potential function [33,34]:

(1) $$E_{LJ}\left(r_{ij}\right)=\frac{C_{12}(i,j)}{{ij}_{12}^{r}}-\frac{C_{6}(i,j)}{{ij}_{6}^{r}}$$

where *r_ij_* is the distance between atoms *i* and *j*, and *C*_6_ and *C*_12_ are the interaction parameters for the attractive and repulsive terms, respectively. For interactions between different atom types, the Lorentz–Berthelot combining rules are applied.

The LJ potential parameters for the main atom types in the PPS molecular chain (carbon C, sulfur S, hydrogen H) are listed in Table 1.

Kevlar (PPTA) molecular chains contain a wider variety of atom types (C, N, O, H, etc.), and their LJ parameters also adopt the standard atom type parameters of the GROMOS 54A7 force field. The LJ parameters used for Kevlar in this study are consistent with those reported in the literature [36].

Regarding simulation parameter settings, this study utilizes the Gromacs 2018 software package. Molecular topologies were generated using the Automated Topology Builder (ATB) [37]. The time step was set to 1.0 fs. The cutoff radius for van der Waals interactions was set to 1.2 nm (12 Å), with atom-based summation employed. Long-range electrostatic interactions were treated using the Particle Mesh Ewald (PME) method [38], with the real-space cutoff consistent with the van der Waals cutoff (1.2 nm). Temperature was controlled using the Nosé-Hoover thermostat [39] with a relaxation time constant of 0.1 ps. In NPT ensemble simulations, pressure was controlled using the Parrinello–Rahman method [40] with a relaxation time constant of 1.0 ps. Periodic boundary conditions were applied in all three dimensions. All bond lengths were constrained using the LINCS algorithm [41]. The convergence criterion for energy minimization was set to a maximum force of less than 10 kJ·mol^−1^·nm^−1^.

### 2.2. PPS Matrix Model Construction

**Figure 1:**
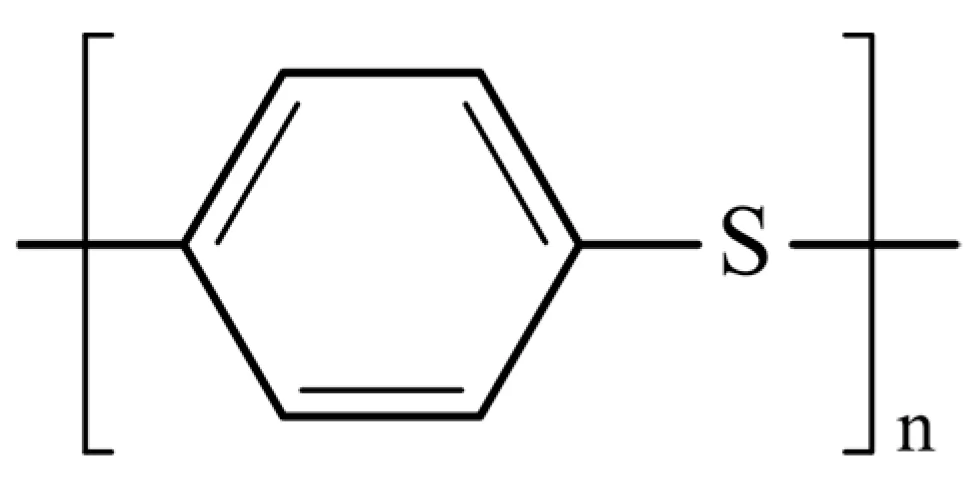
Chemical structure of the PPS repeating unit.

The chemical structure of the repeating unit of polyphenylene sulfide is para-phenylene sulfide (–C_6_H_4_–S–), as shown in Figure 1. The amorphous PPS model constructed in this study contains 50 PPS molecular chains, each consisting of 93 repeating units. The actual degree of polymerization for PPS molecular chains typically ranges from 50 to 150. This study selected 93 repeating units (corresponding to a molecular weight of approximately 10,046 g/mol and a chain length of about 50 nm), a parameter that effectively balances computational accuracy and cost.

**Figure 2:**
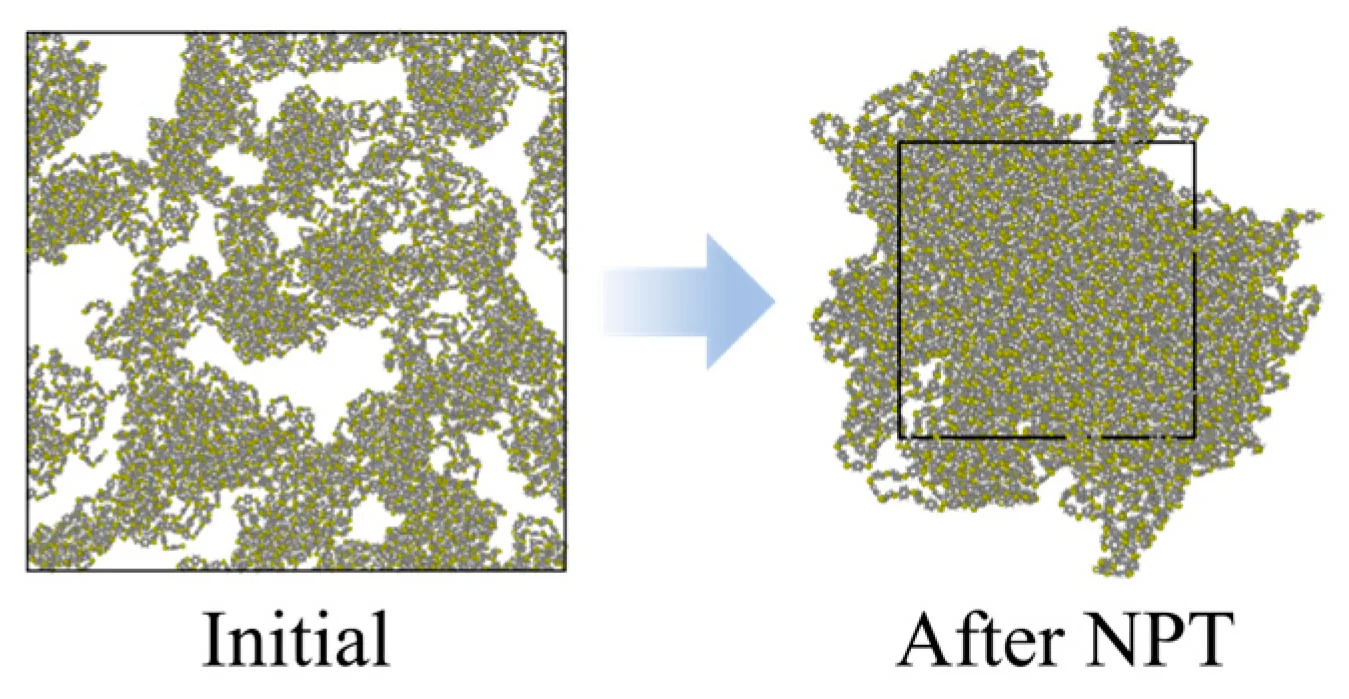
PPS molecular dynamics model before and after relaxation.

The model construction steps were as follows:

(1) Initial Configuration Generation: The initial amorphous configuration was constructed using Gromacs software. The initial model dimensions for the PPS amorphous system were 20 nm × 20 nm × 20 nm, with 50 molecular chains randomly distributed within this space.
(2) Energy Minimization: The initial configuration underwent energy minimization with a convergence criterion of maximum force less than 10 kJ·mol^−1^·nm^−1^ to eliminate unreasonable atomic contacts and overlaps between chains.
(3) NPT Relaxation: An initial relaxation of 1 ns was performed under the NPT ensemble at a pressure of 1 bar and a temperature of 300 K, allowing the system density to converge to the target density of 1.3 g/cm^3^ (close to the actual density of PPS). The relaxed PPS molecular model is shown in Figure 2.

### 2.3. Kevlar Fiber Model Construction

**Figure 3:**
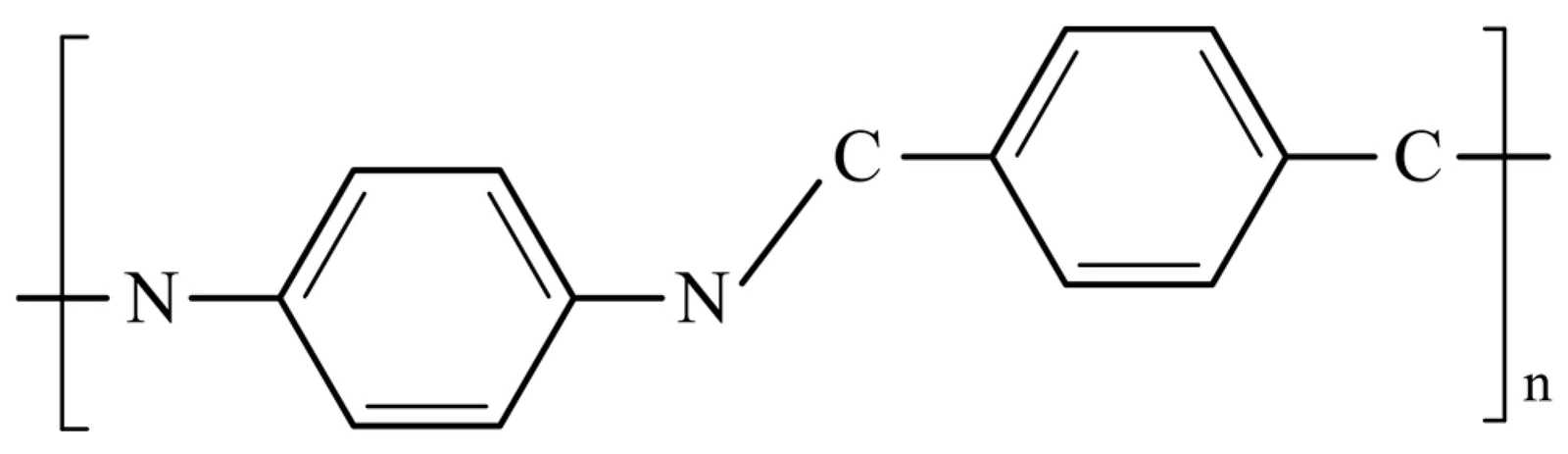
Chemical structure of Kevlar (PPTA) repeating unit.

The repeating unit of Kevlar (PPTA, poly-p-phenylene terephthalamide) is para-phenylene terephthalamide (–NH–C_6_H_4_–NHCO–C_6_H_4_–CO–), as shown in Figure 3. PPTA molecular chains possess extremely high axial rigidity and a regular interchain hydrogen bond network, which are the molecular origins of the high modulus and strength of Kevlar fibers.

The Kevlar fiber model constructed in this study contains 64 molecular chains, each consisting of 6 repeating units. The crystal structure parameters of Kevlar adopted the monoclinic system data reported in the literature [36]: a = 7.87 Å, b = 5.18 Å, c (fiber axis) = 12.9 Å, α = β = γ = 90°.

**Figure 4:**
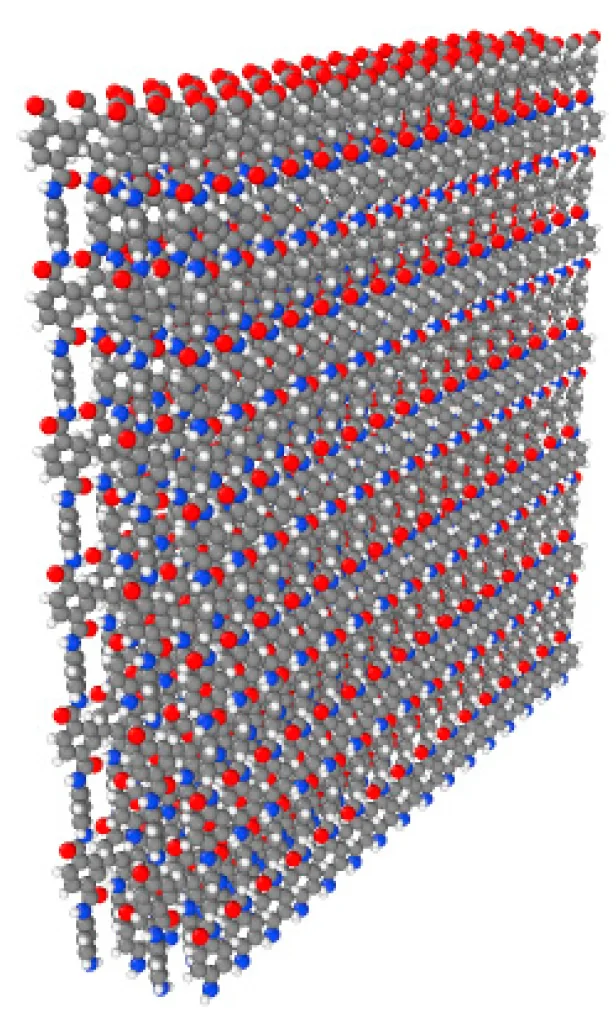
Molecular dynamics model of the Kevlar fiber surface.

The modeling scheme for the PPTA unit cell followed the approach of Wang et al. [36]: the basic building block of the PPTA crystal measured 4.68 nm × 5.19 nm × 5.16 nm and contained poly (p-phenylene terephthalamide) chains regularly arranged along the molecular chain direction. Based on this crystal structure, 64 molecular chains arranged in a 4 × 16 array were adopted to form a crystalline-ordered Kevlar fiber sheet, as shown in Figure 4. The structure is periodic along the molecular chain direction, with the PPTA chains connected end-to-end and therefore possessing no chain ends.

This modeling strategy ensures that the fiber region possesses the correct molecular chain orientation and interchain hydrogen bond network to simulate the Kevlar fiber surface. These hydrogen bond networks are continuously distributed along the fiber direction, endowing Kevlar fibers with extremely high axial modulus and transverse stability. The elastic stiffness matrix (orthotropic material) of the crystal is:

(2) $$C_{core}=\left[\begin{array}{cccccc} 312.90 & 27.03 & 24.46 & 0 & 0 & 0\\ & 73.99 & 37.17 & 0 & 0 & 0\\ & & 47.87 & 0 & 0 & 0\\ & SYM & & 18.36 & 0 & 0\\ & & & & 6.36 & 0\\ & & & & & 16.94 \end{array}\right](GPa)$$

These stiffness matrix data indicate that the axial stiffness of the Kevlar crystal is extremely high (312.9 GPa), but the transverse stiffness is relatively weak (transverse modulus in the core region *C*_22_ ≈ 74 GPa, *C*_33_ ≈ 48 GPa).

### 2.4. PPS/Kevlar Interface Model Construction

**Figure 5:**
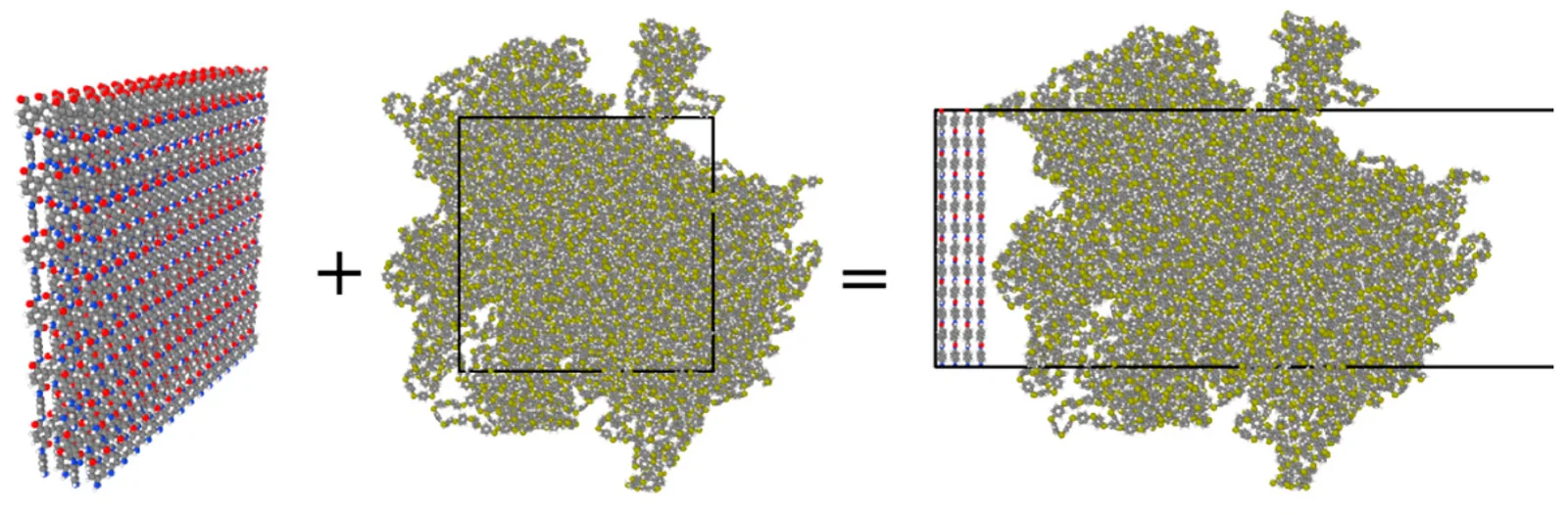
Assembly process of the molecular dynamics model for the Kevlar/PPS composite interface.

The construction of the interface model involved the following key steps:

(1) Assembly: The interfaces of the Kevlar crystal are classified into longitudinal interfaces (planes where molecular chain ends are concentrated) and transverse interfaces (boundaries between adjacent crystals, primarily maintained by hydrogen bonds). The interface between the Kevlar fiber and the PPS polymer matrix is primarily the transverse interface. The equilibrated PPS amorphous model was placed on one side of the Kevlar crystal sheet, with a certain initial spacing maintained between the two phases, and a vacuum layer was reserved on the right side for subsequent tension simulations. The assembled molecular dynamics model is shown in Figure 5.
(2) Energy Minimization: The initial configuration underwent energy minimization with a convergence criterion of maximum force less than 10 kJ·mol^−1^·nm^−1^ to avoid unreasonable configurations where PPS atoms were pushed into the Kevlar lattice.
(3) NVT Relaxation: A 2 ns relaxation simulation was performed under the NVT ensemble to allow PPS molecular chains to fully contact the Kevlar surface through intermolecular interactions and form a stable interfacial structure. As shown in Table 2, temperature control employed an annealing program, subjecting the system to a process from low temperature to high temperature and back to room temperature. This ensured that PPS segments had sufficient kinetic energy to overcome local energy barriers and find low-energy configurations.

### 2.5. Interfacial Normal Tension Simulation

**Figure 6:**
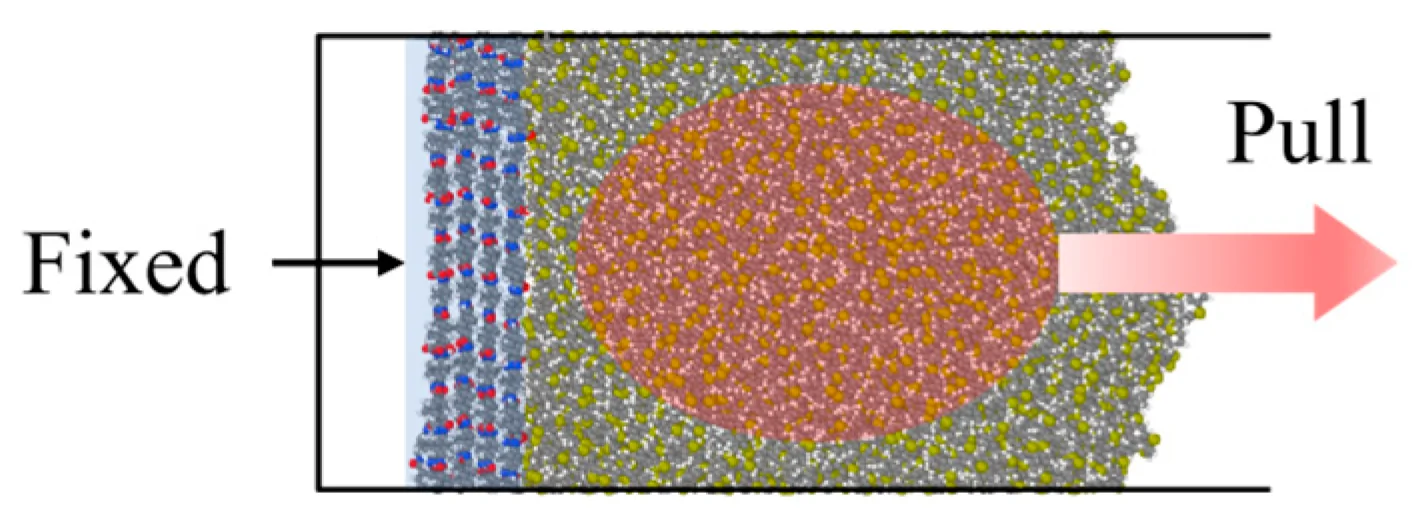
Schematic of the method for simulating interfacial normal tensile strength of Kevlar/PPS composite.

To evaluate the mechanical properties of the interface under normal loading, interfacial normal tension simulations were performed as shown in Figure 6. The specific loading scheme was as follows:

(1) Fixation Strategy: All atoms of the Kevlar layer were fixed to prevent rigid displacement.
(2) Loading Method: All atoms of the PPS layer were selected as pulling points, and a constant-rate tensile load driven by a harmonic potential was applied. The spring constant of the harmonic potential was 0.5 kcal/(mol·Å^2^), ensuring smooth and continuous pulling.
(3) Pulling Rate: 0.1 nm/ns, which falls within the quasi-static tensile loading regime, sufficient to ensure that molecular chains have adequate time for conformational relaxation.
(4) Temperature Condition: 300 K (NVT ensemble).
(5) Data Recording: Atomic coordinates and forces were recorded every 1 ps for subsequent post-processing to determine the stress–strain relationship.

### 2.6. Interfacial Shear Simulation

**Figure 7:**
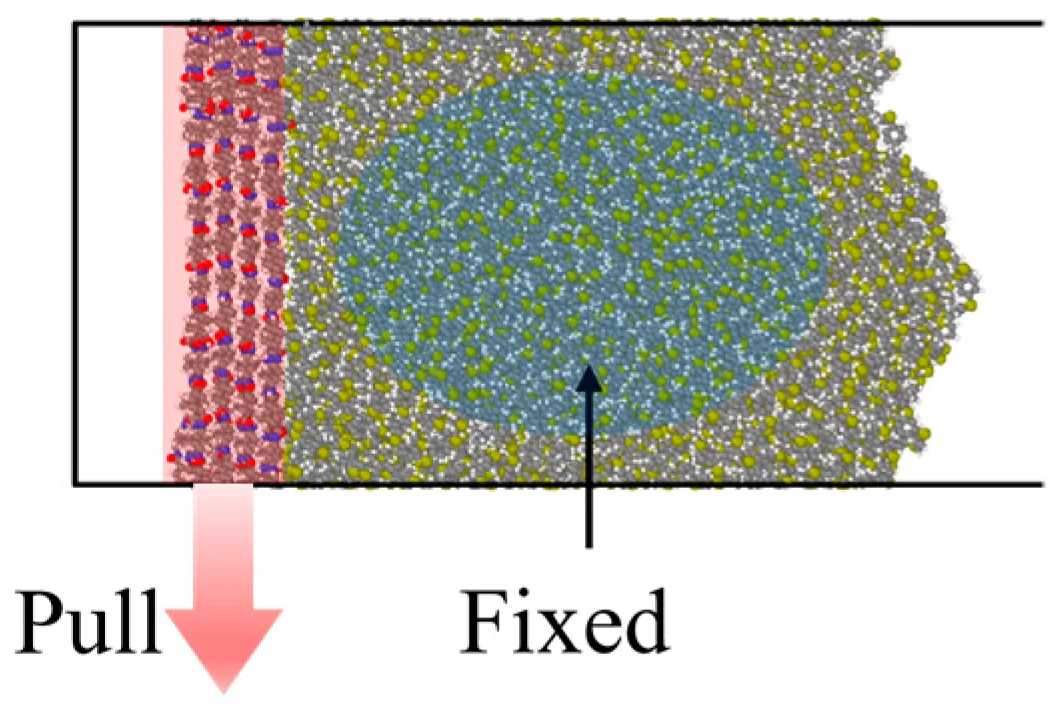
Schematic of the method for simulating interfacial shear strength of Kevlar/PPS composite.

To evaluate the mechanical properties of the interface under shear loading, interfacial shear simulations were performed as shown in Figure 7. The specific loading scheme was as follows:

(1) Fixation Strategy: All atoms of the PPS layer were subjected to fixed constraints.
(2) Loading Method: All atoms of the Kevlar layer were selected, and a harmonic potential-driven traction along the interface direction was applied, causing the Kevlar to undergo shear displacement relative to the PPS.
(3) Pulling Rate: 0.1 nm/ns, consistent with the normal tension simulation.
(4) Temperature Condition: 300 K (NVT ensemble).
(5) Data Recording: Data were recorded every 1 ps.

### 2.7. Control Simulation of Pure PPS

To provide a baseline for comparison with the interfacial strength, a pure amorphous PPS model was independently constructed. This model had parameters identical to the PPS portion in the interface model: 50 molecular chains, each with 93 repeating units, using the same force field, simulation parameters, and annealing procedure. After equilibration, an unconstrained tension simulation was performed to obtain the stress–strain curve of pure PPS as a reference, at a pulling rate of 0.05 nm/ns, consistent with the normal tension rate.

## 3. Results and Discussion

### 3.1. Tensile Experiments of Kevlar/PPS Composites

**Figure 8:**
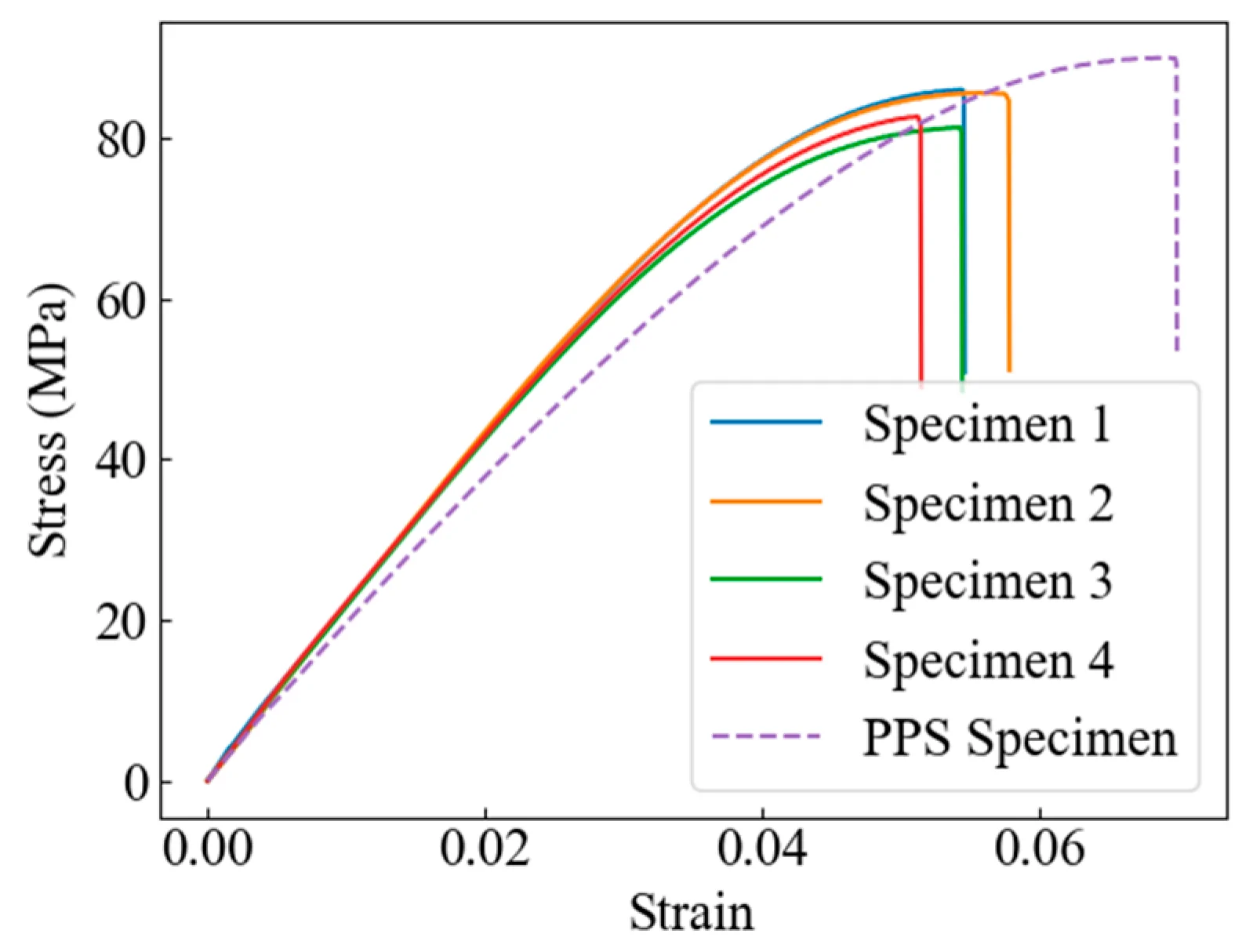
Tensile stress–strain curves of Kevlar/PPS composites.

Tensile test specimens of PPS reinforced with 10 wt% short Kevlar fibers and pure PPS were fabricated by injection molding to compare the reinforcing effect of Kevlar fibers on the PPS matrix. The tensile results are shown in Figure 8 and Table 3.

**Figure 9:**
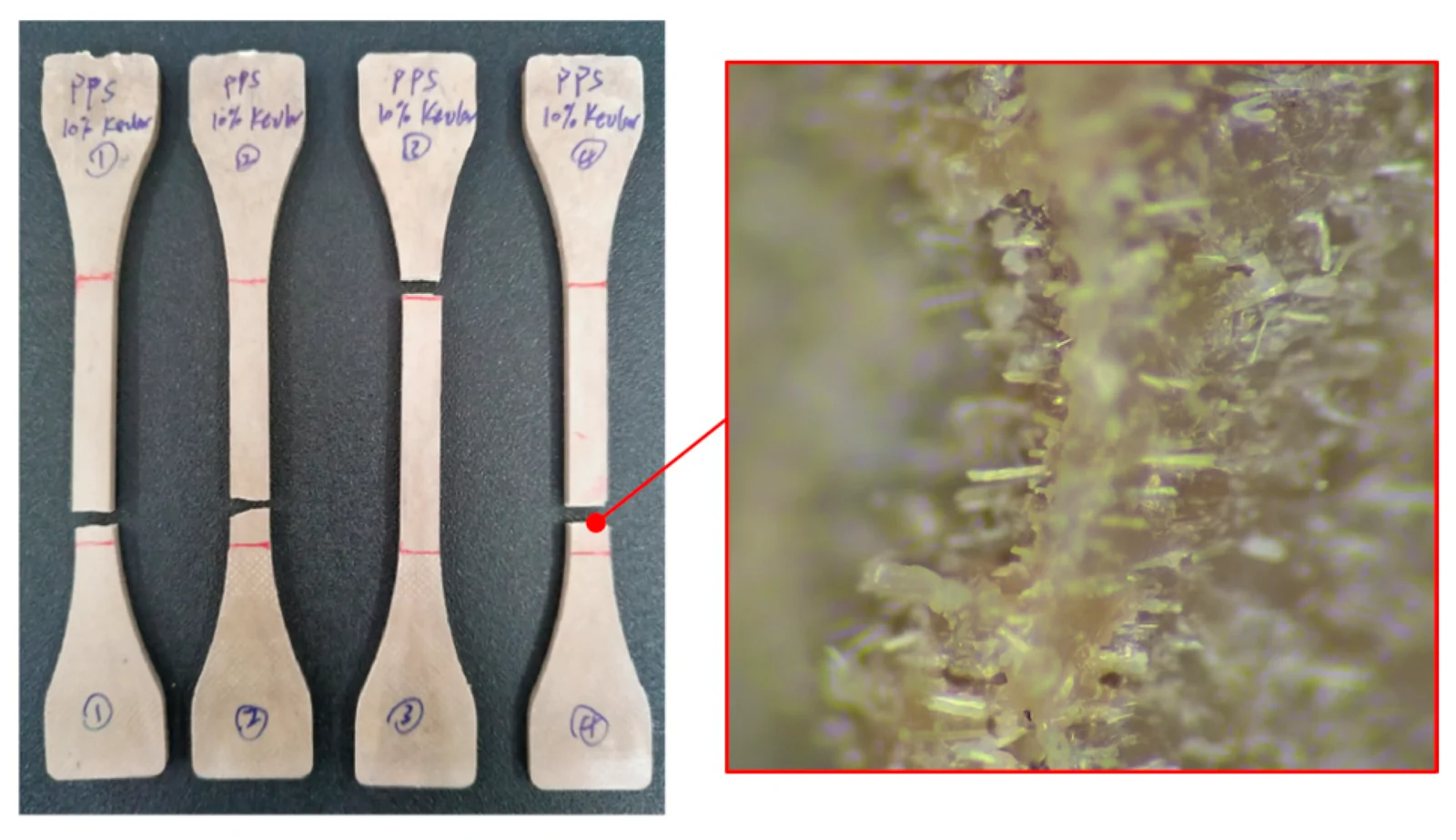
Fracture morphology of Kevlar/PPS tensile specimens.

The comparative tensile experiment results show that the modulus of the Kevlar/PPS composite is higher than that of pure PPS, with an increase of approximately 15.5–22.9%. This indicates that the introduction of Kevlar fibers effectively exploits their high modulus characteristics, constraining the deformation of the PPS matrix through physical constraints during the elastic deformation stage. The strength of the Kevlar/PPS composite decreased by approximately 4.4–9.6% compared to pure PPS, exhibiting the phenomenon of “modulus increase, strength decrease.” The root cause lies in the large elastic modulus mismatch between the fiber and the polymer matrix, which causes the matrix stress near the fiber ends to reach the failure strength first, together with the insufficient interfacial bonding strength that leads to ineffective load transfer. Optical microscopy observation of the fracture surface revealed extensive fiber pull-out traces, with the pulled-out fibers exhibiting smooth surfaces and almost no PPS matrix residue (as shown in Figure 9). This typical brittle interfacial fracture characteristic indicates that the interfacial bonding strength between Kevlar fibers and the PPS matrix is insufficient to achieve effective load transfer.

### 3.2. Preparation of the Interfacial Molecular Dynamics Model

**Figure 10:**
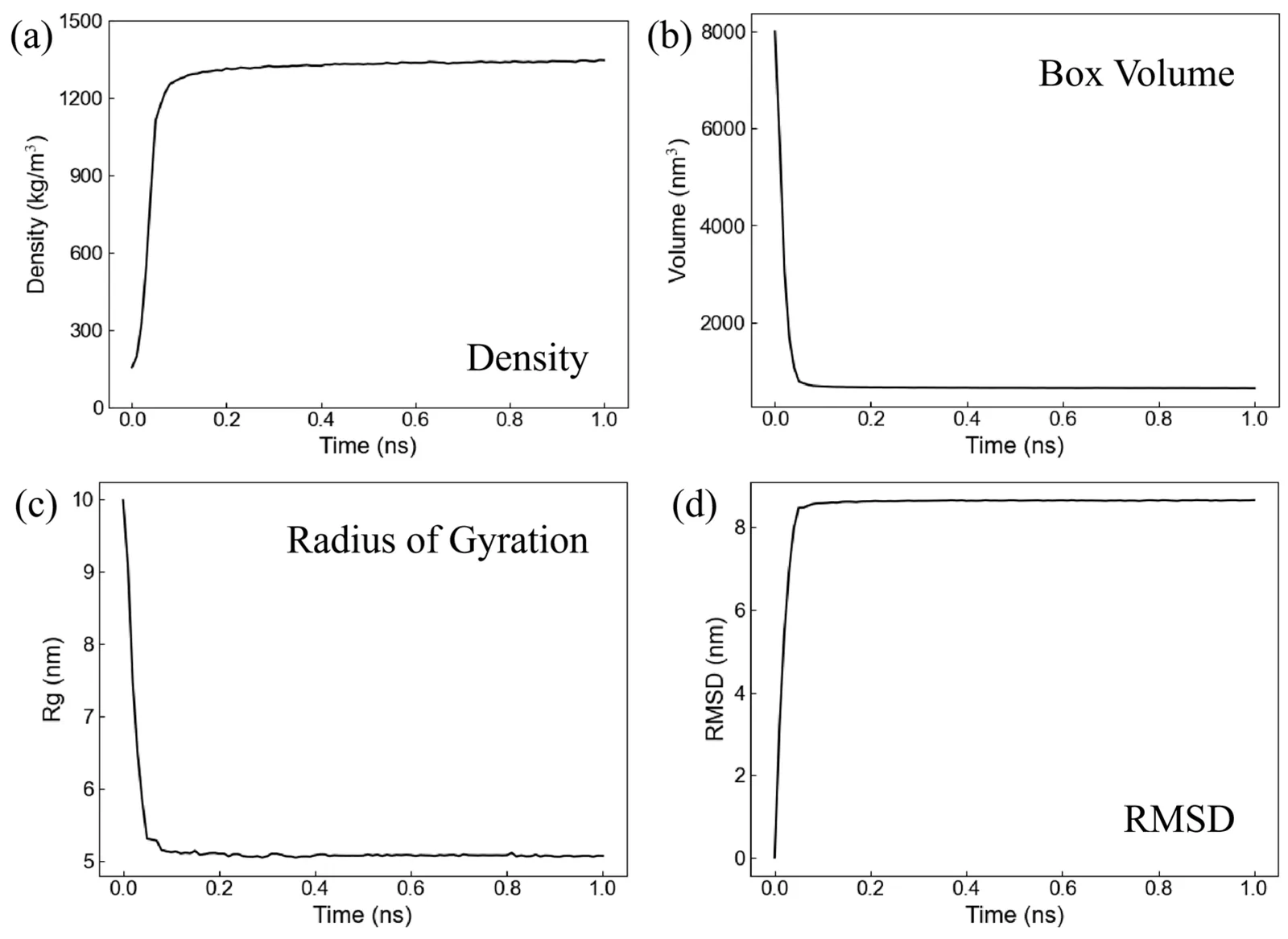
(**a**) Density, (**b**) box volume, (**c**) radius of gyration, and (**d**) RMSD of the PPS system during NPT equilibration.

Before constructing the molecular dynamics model of the PPS/Kevlar interface, the PPS system was first subjected to an independent relaxation treatment, with the final 1 ns performed under the NPT ensemble. Figure 10 presents the evolution of the density, box volume, radius of gyration (Rg), and root-mean-square deviation (RMSD) of the PPS system during this 1 ns period. Within the initial 0.1 ns of the simulation, the system contracted rapidly under the external pressure, and the box volume decreased significantly. During the subsequent 0.9 ns, the molecular chains continuously adjusted their conformations toward an optimal arrangement, and the PPS density eventually stabilized at approximately 1.3 g/cm^3^. The evolution curves of Rg and RMSD indicate that, after 1 ns of NPT relaxation, the PPS molecular chains had essentially reached an equilibrium state.

**Figure 11:**
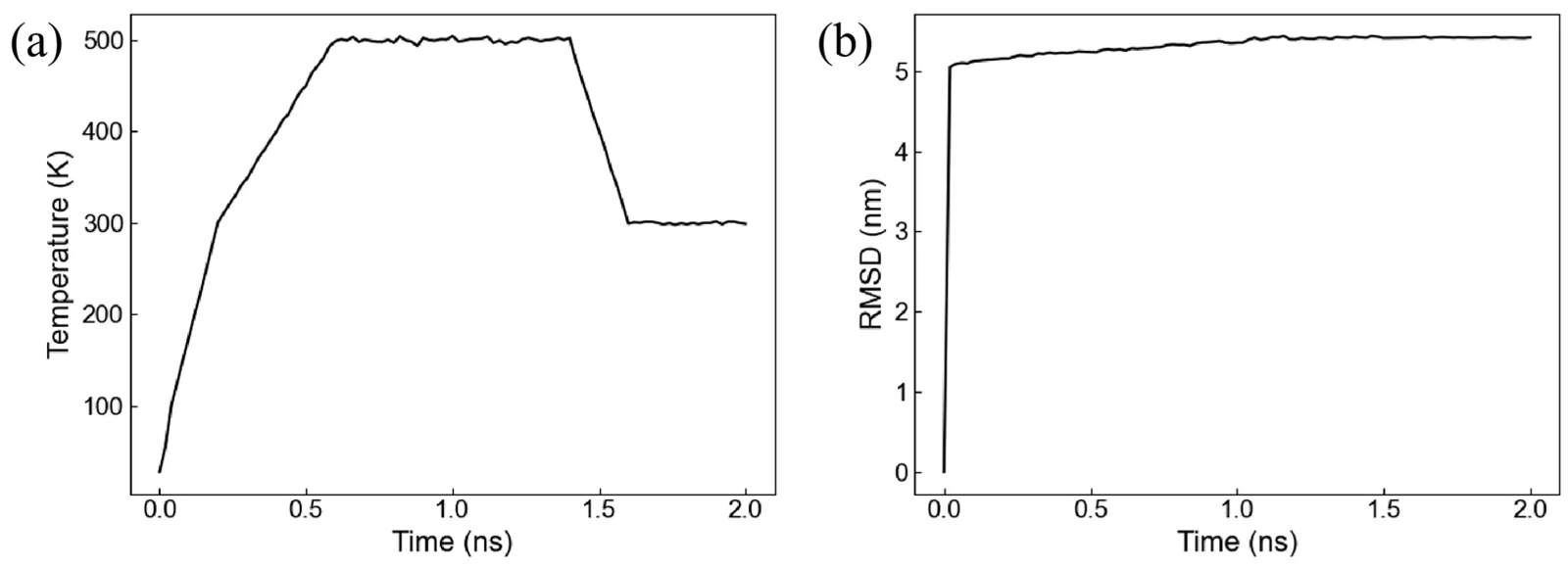
(**a**) Temperature and (**b**) RMSD of the interface system during NVT equilibration.

After the PPS system reached equilibrium via NPT relaxation, the molecular models of PPS and Kevlar were placed in the same simulation box. The box dimension in the X direction (i.e., the normal tension direction) was much larger than those in the other two directions to provide sufficient space for tensile deformation. A certain gap was reserved between PPS and Kevlar so that the two phases could freely adjust to their equilibrium configurations during relaxation. During the subsequent 2 ns NVT relaxation, the evolution of temperature and RMSD of the interface system with time is shown in Figure 11. At the initial stage of NVT relaxation, the RMSD fluctuated significantly, indicating that PPS and Kevlar approached each other driven by non-bonded interactions and the initial gap gradually disappeared. Over the following ~1 ns, the RMSD continued to increase slightly, indicating that the molecular chains of the two phases were adjusting their local conformations. After 1 ns, the RMSD curve leveled off, indicating that the interface system had essentially reached a steady state, and with the temperature gradually decreasing, the interface system eventually converged to equilibrium.

It should be emphasized that the molecular dynamics model employed in this study describes the local chemical interface and short-range non-bonded interactions between PPS and aramid at the molecular level. Macroscopic mechanical phenomena governed by crystallization—such as the transcrystalline layer that may form around fibers in semi-crystalline PPS—are beyond the scope of the current nanosecond-scale all-atom molecular dynamics framework. Therefore, the MD results should be interpreted as providing molecular-level insights into interfacial interactions and failure mechanisms, rather than as direct quantitative predictions of the macroscopic properties of the composite.

### 3.3. Interfacial Normal Tension Behavior

**Figure 12:**
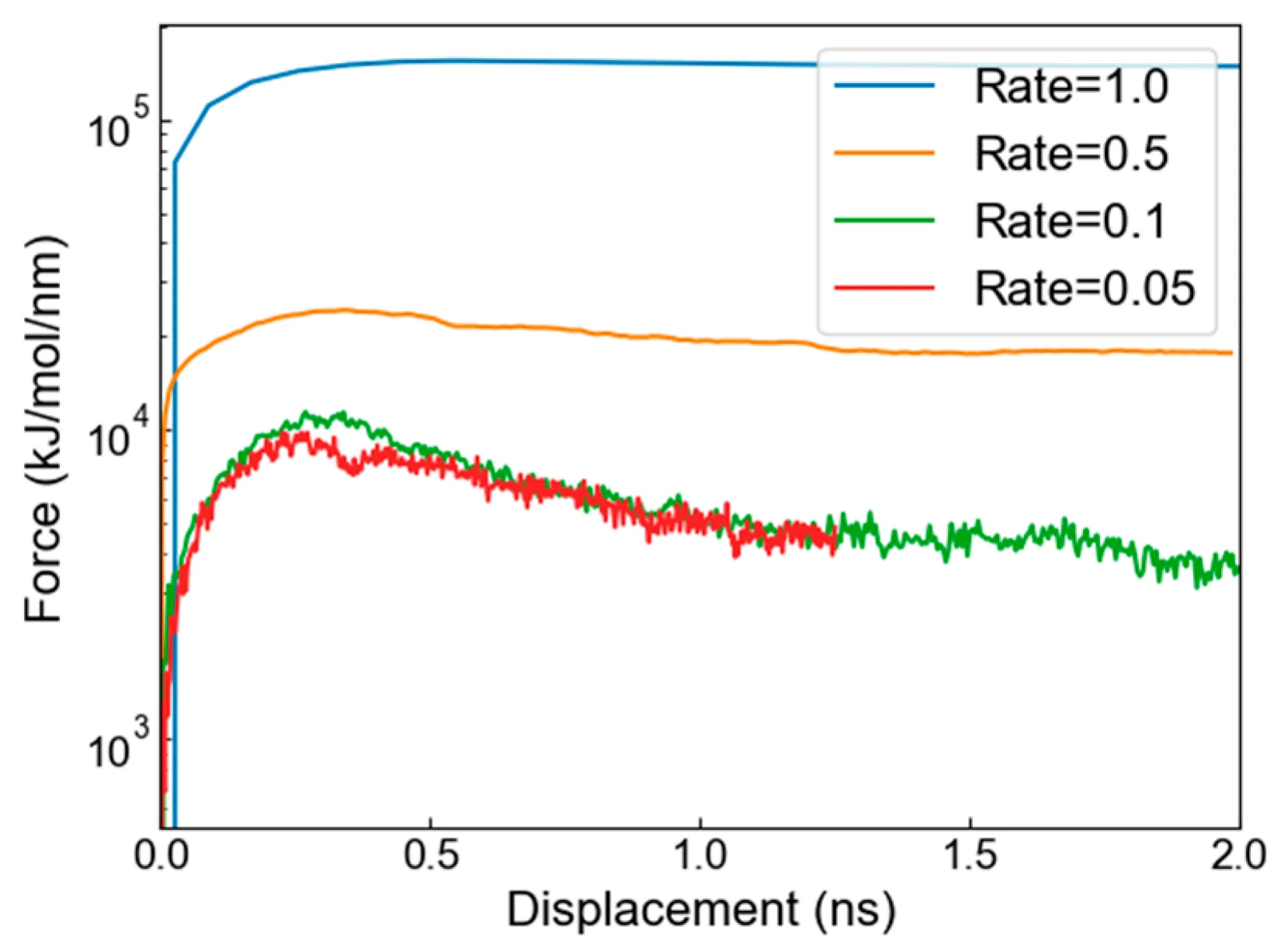
Force-displacement curves of the interface system at different pulling rates.

To better characterize the mechanical properties of the interface, the pulling rate was first discussed, as shown in Figure 12. Four pulling rates, namely 1.0, 0.5, 0.1, and 0.05 nm/ns, were selected for the tension simulations, and the force-displacement relationships at the pulling point were extracted. It can be seen from the curves that the peak force tends to be larger at higher pulling rates, and the force-displacement results are strongly affected by the pulling rate; when the pulling rate is below 0.1 nm/ns, the obtained force-displacement curves show little variation. Therefore, to balance computational efficiency and accuracy, 0.1 nm/ns was selected as the benchmark pulling rate in this study.

To calculate the interfacial strength, the force F needs to be converted into stress via F/A. Since the box is periodic in the Y and Z directions and the Kevlar molecular chains are connected end-to-end in the Z direction, the interfacial cross-sectional area A is the area of the box perpendicular to the tension direction, i.e., the product of the Y and Z dimensions of the box.

**Figure 13:**
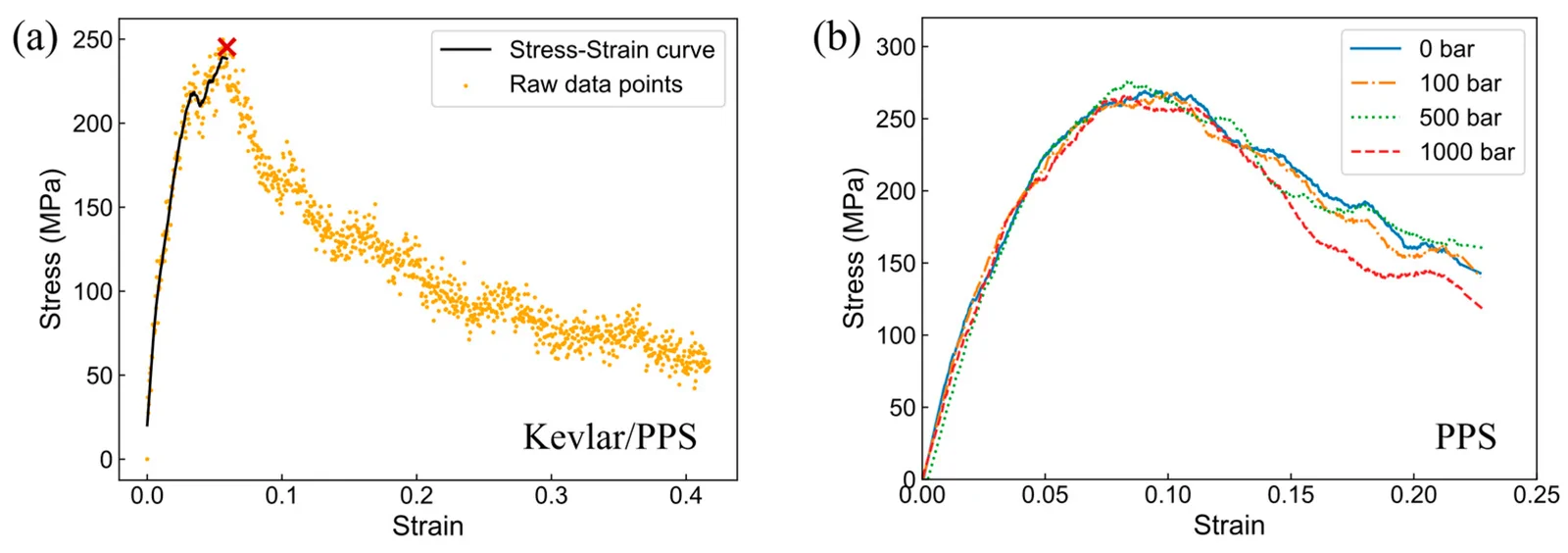
Tensile stress–strain curves for (**a**) Kevlar/PPS interface normal direction and (**b**) pure PPS.

The stress–strain curve obtained from the interfacial normal tension simulation is shown in Figure 13a, where the red x marks represent the points of interface failure. Figure 13b presenting the tensile curve of pure amorphous PPS for comparison. Prior to tension simulation, the pure amorphous PPS was subjected to relaxation under the NPT ensemble at four different pressures to alter its molecular free volume. From the curves in Figure 13b, it can be observed that the different relaxation pressures had a minor effect on the tensile mechanical properties of amorphous PPS, which also eliminated some numerical interference factors in the simulation of the Kevlar/PPS interfacial mechanical properties.

It should be noted that the stresses of PPS obtained from the MD simulations (Figure 13) are much higher than the experimentally measured values (Figure 8). This quantitative discrepancy originates from several fundamental differences between the MD simulation system and the experimental system. First, the strain rate in the MD simulations is several orders of magnitude higher than that in macroscopic experiments. Second, the MD model corresponds to a defect-free amorphous PPS structure, whereas voids and other defects exist in the experimental specimens. In addition, the MD simulation system has a small size and adopts periodic boundary conditions, which limits the length scale over which stress can be redistributed. All of these factors contribute to the differences between the MD simulation results and the experiments. Therefore, the MD stress–strain data should be regarded as a qualitative/relative benchmark for comparing interfacial mechanisms, rather than a direct quantitative prediction of the experimental tensile properties.

**Figure 14:**
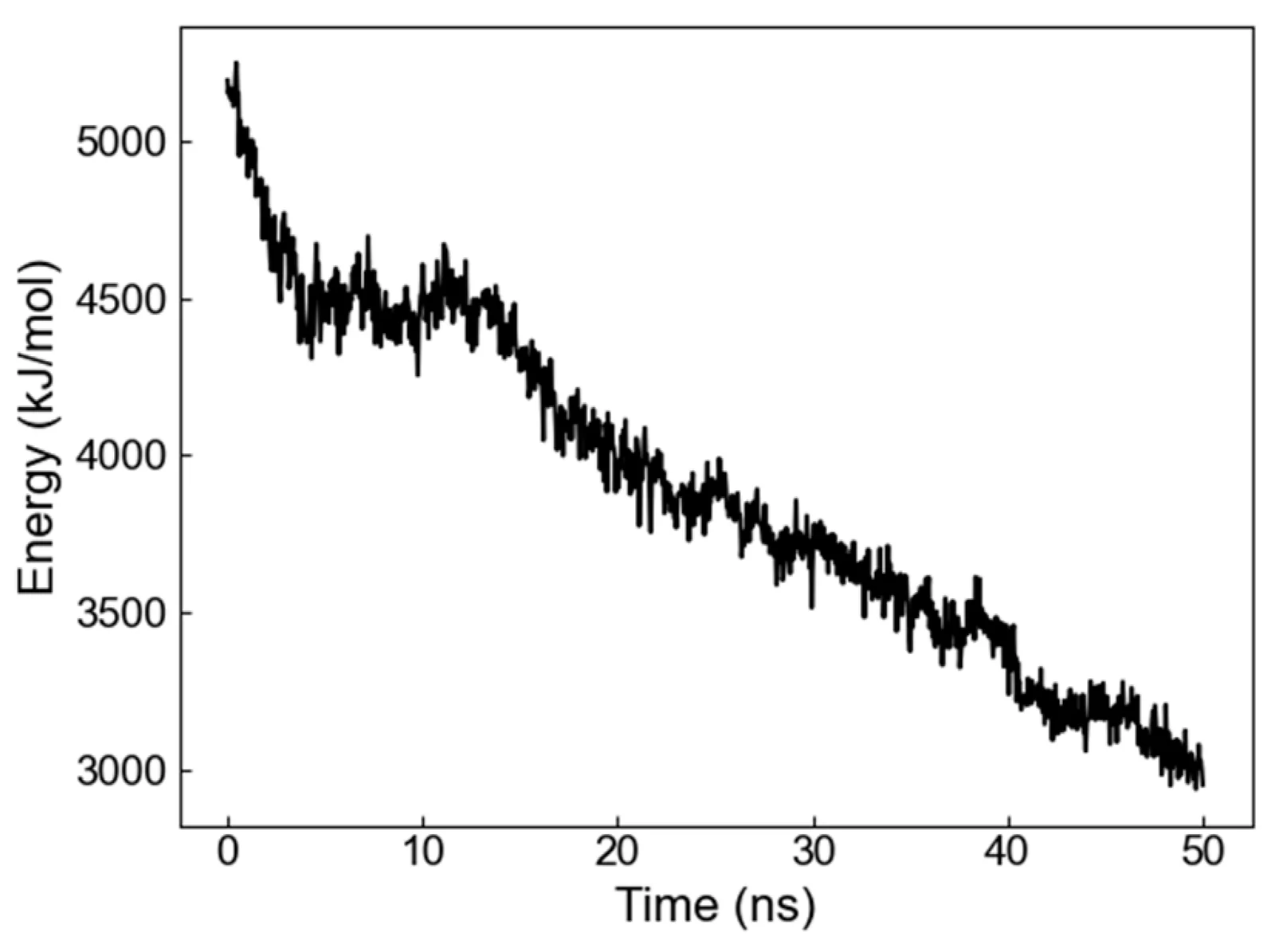
Binding energy between PPS and Kevlar during interfacial normal tension.

The evolution of the binding energy between PPS and Kevlar during the normal tension process is shown in Figure 14. The binding energy includes the LJ potential energy and Coulombic interactions. It can be seen from the figure that, as the tension proceeds, the binding energy decreases rapidly and fluctuates at the point corresponding to the peak stress, with several small rebounds, after which it declines with a gentler trend than in the initial stage.

**Figure 15:**
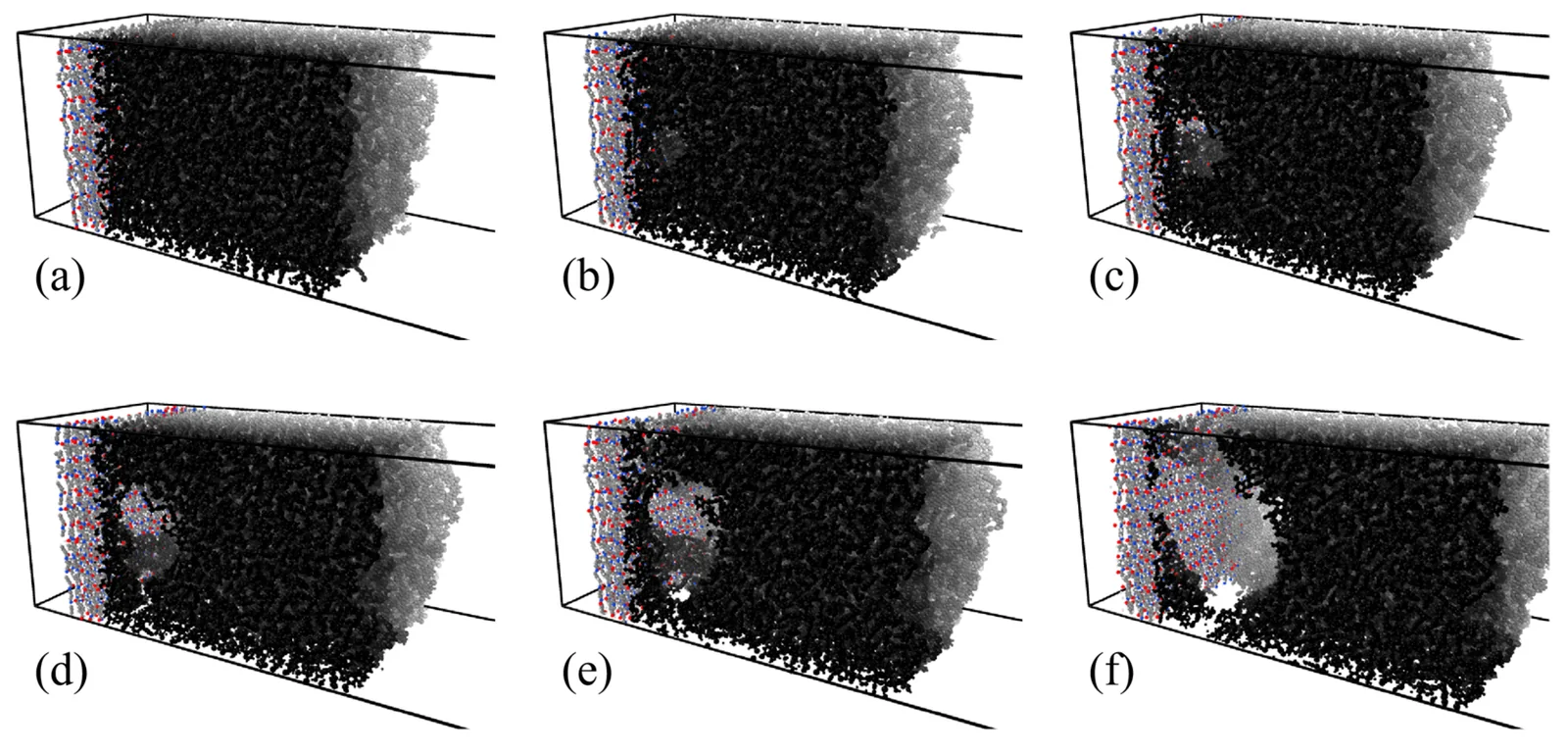
Failure evolution of the Kevlar/PPS interface during normal tension, with strains of (**a**) 0, (**b**) 0.03, (**c**) 0.06, (**d**) 0.12, (**e**) 0.18, (**f**) 0.36 respectively.

The interfacial normal tension curve exhibits elastic–plastic response characteristics, which can be divided into an elastic region, a yield region, and a failure region by correlating the tensile curves in Figure 13 with the molecular model snapshots at different stages in Figure 15, and rendering the PPS molecular chains in black for better visualization of the interfacial failure evolution.

In the elastic region (strain < 3%), stress increases linearly with strain, with reversible deformation of bond lengths and bond angles dominating. The response of the interfacial system in this stage is similar to that of pure PPS, indicating that the initial stiffness is primarily contributed by the PPS matrix. However, the tensile modulus of the interface is slightly higher than that of pure PPS, as the modulus of the Kevlar layer is much higher than that of PPS, increasing the composite system’s overall modulus.

Upon entering the nonlinear yield region (strain of 3–6%), the curve begins to fluctuate, and voids start to form between the PPS layer and the aramid layer near the interface. The peak stress is approximately 245 MPa (corresponding to a strain of about 6%), after which the stress enters the descending stage. Compared with the peak tensile stress of pure amorphous PPS (approximately 270 MPa), the interfacial normal strength (approximately 245 MPa) is significantly lower, with a reduction of about 9.3%. By tracking the molecular configuration evolution, the failure initiation point is located near the geometrical interface where PPS is in direct contact with Kevlar, and a small number of PPS molecular chains remain attached after separation.

The interlayer interfacial strength of the Kevlar crystal simulated with a similar molecular configuration exceeds 1500 MPa [36], far greater than the strengths of PPS and the interface. It is therefore concluded that damage first initiates on the PPS side, starting from the interface and gradually propagating into the PPS matrix, causing part of the matrix to separate from the bulk. In addition, it was observed from the MD trajectories that voids form preferentially near the geometrical interface rather than inside the PPS matrix, which can be reasonably attributed to the introduction of Kevlar fibers, which induces an abrupt change in the mechanical properties, such as the tensile modulus, at the interface—generally the fundamental reason why the interface is prone to damage.

The molecular models during the normal tension process also explain the evolution trend of the binding energy. At the initial stage of tension, the distance between the PPS and Kevlar molecular chains gradually increases and the interfacial gap widens overall, corresponding to the linear elastic stage in which the stress increases while the binding energy decreases linearly. When the peak stress is reached, local PPS molecular chains begin to diffuse from the interface toward the interior and both sides of the PPS, causing the PPS chains on both sides to move slightly closer to the Kevlar chains; at this point, the binding energy rebounds slightly, while the stress decreases because the interfacial gap and contact area are reduced. Subsequently, the distance between the PPS bulk and the Kevlar molecular chains continues to increase, and the binding energy resumes a linear decrease; however, some PPS molecular chains peel off from the bulk and adhere to the Kevlar surface, reducing the rate of the binding energy decline.

The potential influence of the fully fixed constraint of Kevlar on the above conclusions warrants the following discussion. In the normal tension simulations of this study, the Kevlar atoms were completely fixed, meaning that the PPS chain segments at the interface experienced a perfectly rigid surface. In reality, the atoms on the Kevlar surface are not completely immobile; in particular, the molecular chains near the fiber surface may undergo local out-of-plane displacement (albeit very small in magnitude) under the adhesion of PPS, which would alter the stress distribution at the interface and typically delay the onset of damage. However, considering that the transverse modulus of the Kevlar crystal exceeds 100 GPa [36], far higher than that of PPS, the changes in mechanical properties caused by such local surface flexibility are relatively limited and would not alter the conclusions drawn from the above discussion.

### 3.4. Interfacial Shear Behavior

**Figure 16:**
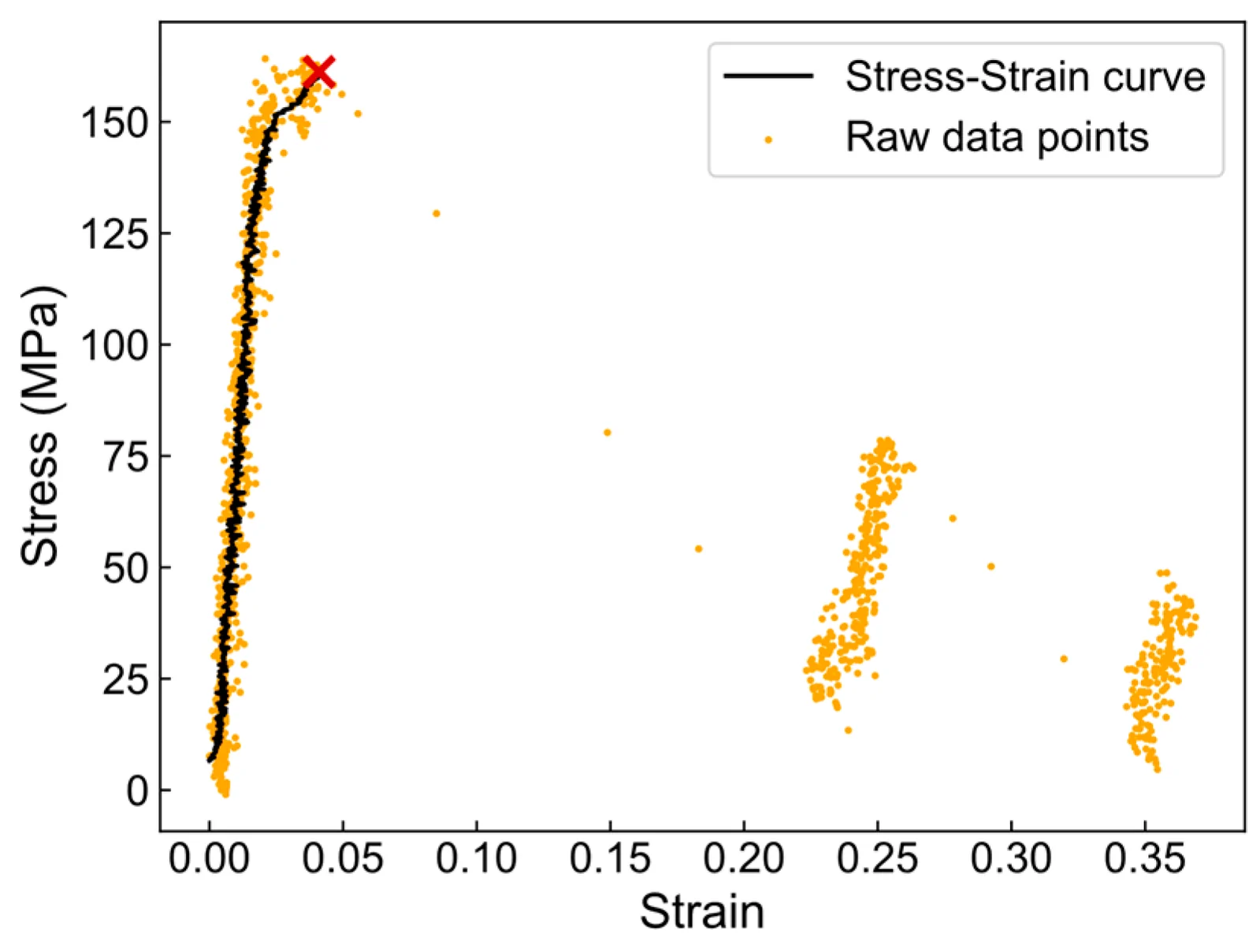
Interfacial shear stress–strain curve.

**Figure 17:**
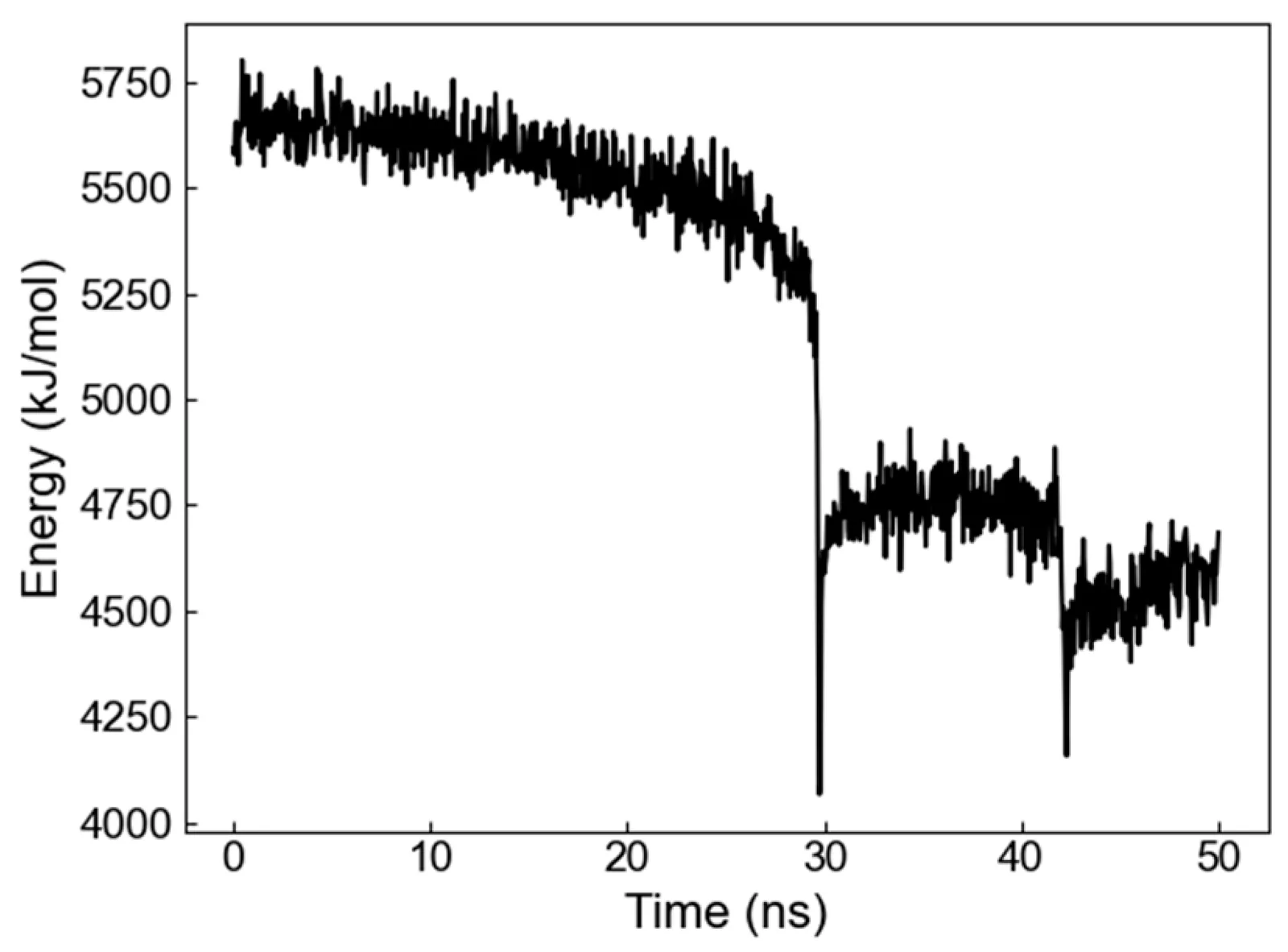
Binding energy between PPS and Kevlar during interfacial shear.

**Figure 18:**
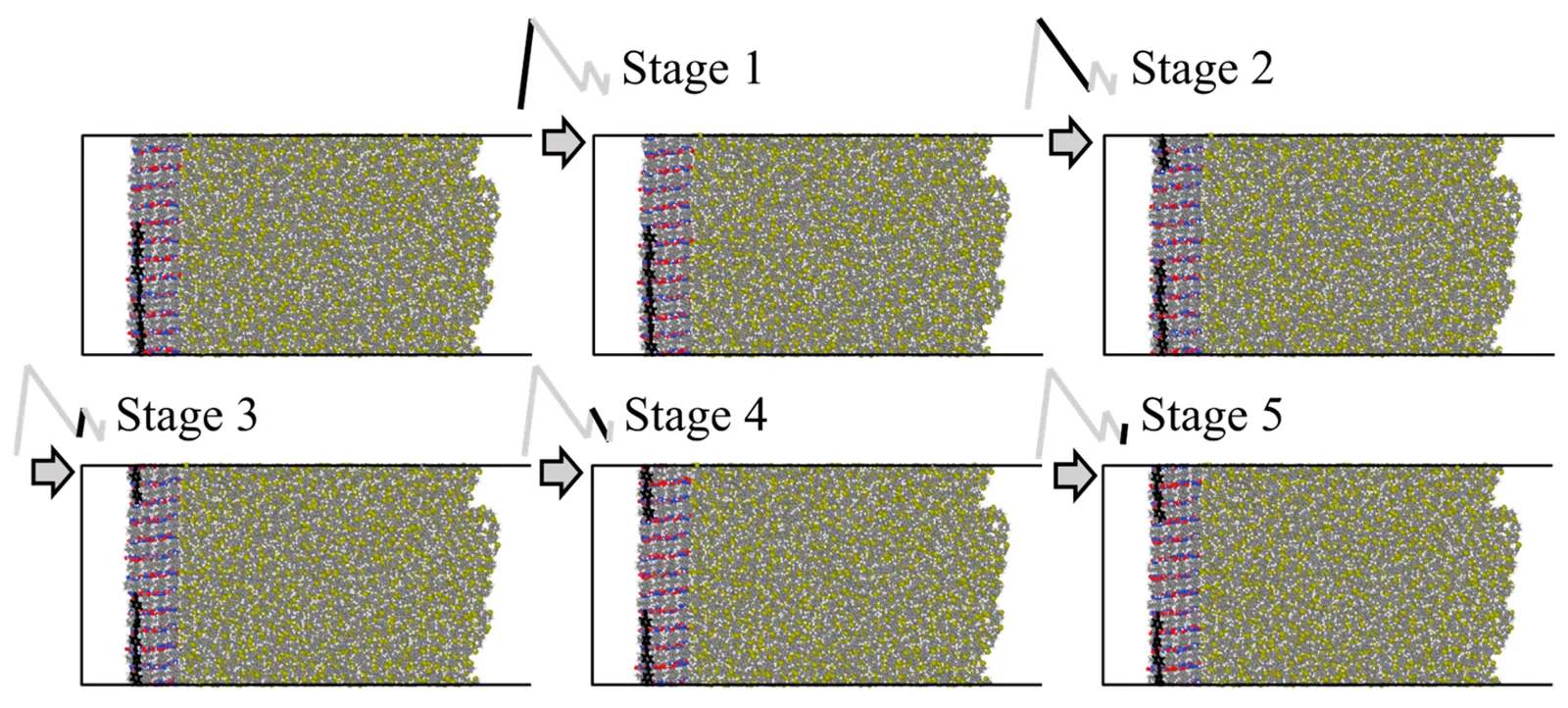
Schematic of the five-stage damage evolution during interfacial shear.

The interfacial shear simulation reveals a more complex multi-stage damage evolution process than normal tension. The stress–strain curve exhibits serrated stick-slip characteristics, as shown in Figure 16, where the red x marks represent the interface failure points. The evolution of the binding energy between PPS and Kevlar during tangential shear is shown in Figure 17. Combined with visual analysis of the molecular configuration evolution, half of the Kevlar molecular chains were rendered in black to facilitate observation of relative displacement, and the shear process was divided into five stages, as illustrated schematically in Figure 18.

The molecular mechanisms for each stage are analyzed in detail below:

(1) Stage 1: At relatively small shear strains (strain < 3%), the PPS and Kevlar on both sides of the interface undergo uniform elastic shear deformation. The bond angles and dihedral angles of the PPS molecular chains undergo reversible deformation, with no significant change in the overall molecular chain conformation. The shear stress and shear strain exhibit a linear relationship, and accordingly, the binding energy also decreases linearly.
(2) Stage 2: When the shear stress reaches a critical value (the first peak of the curve, approximately 165 MPa), localized relative slide begins at the interface. By rendering half of the Kevlar molecular chains in black, the localized sliding of the Kevlar fiber relative to the PPS matrix can be clearly observed. After localized sliding occurs, the stress drops rapidly. At this point, the binding energy also exhibits a sudden decrease, which is likely because the sites with strong non-bonded interactions between the Kevlar and PPS molecular chains rupture as a whole; i.e., an overall sliding of the two phases occurs.
(3) Stage 3: The PPS chain segments in the sliding region re-establish relatively strong non-bonded interactions with the aramid molecular chains at the interface, and the stress accumulates again. Meanwhile, the binding energy also rises abruptly after the sudden drop.
(4) Stage 4: When the accumulated stress is sufficient to overcome the intermolecular forces, sliding occurs again at the interface, and stress is rapidly released. This stick-slip process occurs alternately across the entire interface, manifesting as serrated fluctuations in the stress–strain curve.
(5) Stage 5: As the shear displacement continues to increase, the amplitude of the stick-slip cycles gradually attenuates, interfacial damage continuously accumulates, and ultimately the Kevlar fiber completely debonds from the PPS matrix.

The stress–strain evolution of interfacial shear exhibits typical stick-slip behavior, which is essentially analogous to the “static friction–dynamic friction” transition at the atomic scale, representing the macroscopic manifestation of the periodic overcoming and re-establishment of intermolecular forces between the Kevlar surface and PPS segments.

### 3.5. Prospects for Interface Modification

Based on the molecular-scale mechanistic understanding obtained in this study and the research of other scholars, the following targeted modification strategies for improving the interfacial properties of Kevlar/PPS composites are summarized. Mainstream approaches include plasma treatment, chemical grafting, sizing agent coating, and other technical routes.

(1) Kevlar Fiber Surface Treatment: Introducing polar oxygen-containing functional groups onto the Kevlar fiber surface through oxygen plasma or air plasma treatment to enhance dipole interactions with PPS sulfur atoms [11,42].
(2) Local Structural Regulation of the Matrix: Incorporating nanofillers such as carbon nanotubes and graphene [43,44] to construct a three-dimensional network structure in the interfacial region, modulating load transfer and interfacial stress distribution in the composite interface region.
(3) Interfacial Layer Design: Constructing an intermediate layer with a graded stiffness gradient on the Kevlar fiber surface. For example, by in-situ grafting of polyurea and infiltrating epoxy resin on the fiber surface, a “rigid-and-soft” interlocking three-dimensional interphase structure can be formed to enhance interfacial shear strength and toughness [45].

## 4. Conclusions

In this study, a full-atom molecular dynamics interface model between Kevlar fibers and the PPS matrix was constructed, and the interfacial mechanical response and damage evolution mechanisms of the Kevlar/PPS composite system at the molecular scale were systematically revealed through normal tension and tangential shear simulations. Comparative analysis with pure PPS and the Kevlar crystal indicates that the interfacial normal strength is limited by the modulus mismatch between the PPS matrix and the rigid fiber surface. This study clearly distinguishes two distinct failure modes under interfacial normal and tangential loading in the Kevlar/PPS system and summarizes the corresponding molecular-level damage criteria. Under normal tension, voids preferentially form in the matrix near the interface and propagate into the PPS interior, exhibiting brittle fracture characteristics; under tangential shear, the interface exhibits a serrated stick-slip response. These atomistic mechanistic interpretations provide modification directions for the regulation of interfacial stiffness gradients and surface polarity modification. The simulation methods established and the knowledge framework obtained in this study can also be extended to the interfacial design and performance optimization of other aromatic thermoplastic composite systems, providing a valuable reference for the multiscale forward development of high-performance composites.

## Figures and Tables

**Table 1:** Lennard-Jones 12-6 potential parameters for PPS [35].

Atom Type	Atomic Mass (g/mol)	*C*_6_ (kJ·mol^−1^·nm^6^)	*C*_12_ (kJ·mol^−1^·nm^12^)
C	12.01	2.341 × 10^−3^	4.937 × 10^−3^
S	32.07	9.984 × 10^−3^	1.307 × 10^−3^
H	1.0	8.464 × 10^−3^	1.513 × 10^−3^

**Table 2:** Temperature settings for NVT ensemble simulation of Kevlar/PPS composite.

Time (ps)	Temperature (K)	Stage Description
0	10	Initial ultra-low temperature
40	100	Slow heating
200	300	Heating to room temperature
600	500	Heating to high temperature
1400	500	High temperature holding to promote segmental motion
1600	300	Cooling to room temperature
2000	300	Holding at room temperature to completion

**Table 3:** Tensile modulus and tensile strength of Kevlar/PPS composites.

Specimen	Tensile Modulus (MPa)	Tensile Strength (MPa)
Specimen 1	2181.59	85.97
Specimen 2	2287.81	85.59
Specimen 3	2278.77	81.28
Specimen 4	2322.35	82.64
PPS Specimen	1889.18	89.93

## Data Availability

The data presented in this study are available on request from the corresponding author.

## Abbreviations

PPS	Polyphenylene Sulfide
PPTA	Poly(p-phenylene terephthalamide)
MD	Molecular Dynamics
LJ	Lennard-Jones
NVT	Constant Number of Particles, Volume, and Temperature
NPT	Constant Number of Particles, Pressure, and Temperature
PME	Particle Mesh Ewald
ATB	Automated Topology Builder
Tg	Glass Transition Temperature
Tm	Melting Temperature
